# Biarticular mechanisms of the gastrocnemii muscles enhance ankle mechanical power and work during running

**DOI:** 10.1098/rsos.230007

**Published:** 2023-08-30

**Authors:** Adamantios Arampatzis, Mohamadreza Kharazi, Christos Theodorakis, Falk Mersmann, Sebastian Bohm

**Affiliations:** ^1^ Department of Training and Movement Sciences, Humboldt-Universität zu Berlin, Philippstr. 13, Haus 11, 10115 Berlin, Germany; ^2^ Berlin School of Movement Science, Humboldt-Universität zu Berlin, 10115 Berlin, Germany

**Keywords:** energy transfer, energy redistribution, Achilles tendon force, energy storage and recoil

## Abstract

The objective of the study was to explore how biarticular mechanisms of the gastrocnemii muscles may provide an important energy source for power and work at the ankle joint with increasing running speed. Achilles tendon force was quantified as a proxy of the triceps surae muscle force and the contribution of the monoarticular soleus and the biarticular gastrocnemii to the mechanical power and work performed at the ankle joint was investigated in three running speeds (transition 2.0 m s^−1^, slow 2.5 m s^−1^, fast 3.5 m s^−1^). Although the contribution of the soleus was higher, biarticular mechanisms of the gastrocnemii accounted for a relevant part of the performed mechanical power and work at the ankle joint. There was an ankle-to-knee joint energy transfer in the first part of the stance phase and a knee-to-ankle joint energy transfer during push-off via the gastrocnemii muscles, which made up 16% of the total positive ankle joint work. The rate of knee-to-ankle joint energy transfer increased with speed, indicating a speed-related participation of biarticular mechanisms in running. This energy transfer via the gastrocnemii seems to occur with negligible energy absorption/production from the quadriceps vasti contractile elements and is rather an energy exchange between elastic structures.

## 1. Introduction

The ankle joint is a key source of mechanical power and work during human running [1,2]. Inverse dynamic approaches show that the ankle joint mechanical power and work increase with running speed [3,4]. Although ankle joint moment, power and work calculated using inverse dynamics are the result of all forces acting around the ankle joint (i.e. forces of synergist and antagonist muscles, forces transmitted by ligaments, bone-to-bone contact forces and forces from soft tissues around the ankle joint), the importance of the triceps surae muscles, as the main plantar flexors, for the power and work performed at the ankle joint during running is well accepted [5–7]. As the most voluminous of the triceps surae muscles, the soleus generates the greatest part of the moment, power and work at the ankle joint during running [6,7]. The biarticular gastrocnemii muscles generate moments in both the ankle and knee joint and thus redistribute the performed musculotendinous power and work over the two spanned joints [8–10]. Biarticular muscles may also transfer power and energy from the more proximal monoarticular muscles to distal joints and, therefore, may regulate the redistribution and transfer of mechanical power and energy between the spanned joints to be effective at the joint where they are required [8,10].

There are two functional conditions where the unique capability of the gastrocnemii muscles to generate moments simultaneously at the ankle and knee joint, can affect the mechanical power and work at the ankle joint, independent of their own musculotendinous power and work production [9–11]. First, opposite signs in the mechanical power of the gastrocnemii muscles at the ankle and knee joints lead to an energy transfer between the two joints. Second, same signs in the mechanical power of the gastrocnemii muscles at the two joints indicate a simultaneous energy absorption/production of the gastrocnemii muscles at the ankle and knee joint, which leads to a redistribution of their musculotendinous power and work. The definitions of all possible scenarios of energy transfer and energy distribution of the biarticular gastrocnemii muscles at the ankle and knee joints are described in detail by Prilutsky *et al*. [12]. Musculoskeletal models predict a relevant energy transfer from knee to ankle joint via the biarticular gastrocnemii muscles during the push-off phase of jumping and accelerated sprinting. The predicted knee-to-ankle joint energy transfer was reported to be 23 to 25% of the total positive mechanical work performed at the ankle joint during maximum vertical jumping [9,13] and 28% during accelerated sprint running [14]. During steady state submaximal running, where the metabolic cost of transport is more important than maximum power output, information concerning the contribution of biarticular mechanisms to the mechanical power and work at the ankle joint is scarce.

Contractile work of the more proximal and, compared to the triceps surae, more voluminous monoarticular quadriceps vasti [15,16] increases the ankle joint power and work during explosive high intensity tasks through a knee-to-ankle joint energy transfer via the biarticular gastrocnemii [9,13,14]. Due to their longer fibres, however, a unit of force generation is metabolically more expensive compared to the shorter soleus and gastrocnemii muscles [17–19] and, therefore, the knee-to-ankle joint energy transfer mechanism may be less advantageous in submaximal running. One study that investigated the transfer of mechanical energy via the gastrocnemii muscles at a very low running speed (i.e. 1.78 m s^−1^) reported a knee-to-ankle joint energy transfer of 8% of the mechanical work at the ankle [9]. Humans switch voluntarily from walking to running at speeds around 2.0 m s^−1^ [20]. Therefore, the investigated speed of 1.78 m s^−1^ is rather unusual for running and the reported energy transfer between ankle and knee joint might not be representative for running. Thus, the function of the biarticular gastrocnemii muscles at the ankle and knee joint during higher and more physiological running speeds and the possible modulation of biarticular mechanisms to enhance power and work production at the ankle joint with increasing running speed are currently unknown.

In the current study, the Achilles tendon (AT) elongation during running at three speeds (transition 2.0 m s^−1^, slow 2.5 m s^−1^ and fast 3.5 m s^−1^) was measured and the corresponding AT force and AT elastic energy storage and recoil were quantified based on an individually determined AT force–elongation relationship as a calibration measure. Further, based on the AT force as a proxy of the triceps surae muscle force, the generated moment and the performed power and work of the monoarticular soleus muscle at the ankle joint and the biarticular gastrocnemii at the ankle and knee joint were calculated. The main objective was to gain a better understanding of how the monoarticular soleus and the biarticular gastrocnemii contribute to the mechanical power and work performed at the ankle joint and how biarticular mechanisms of the gastrocnemii could provide an important energy source for the increase of power and work at the ankle joint with increasing running speed. It was expected that the soleus muscle would show a greater contribution to the performed ankle joint power and work compared to the gastrocnemii, due to the higher volume of the soleus. However, it was also hypothesized that a knee-to-ankle joint energy transfer via the gastrocnemii muscles promotes the increase of mechanical power and work at the ankle joint during the push-off phase of running.

## 2. Methods

### 2.1. Participants and experimental design

Thirteen healthy individuals (four female) participated in the study (age 28 ± 4 years, body height 175.0 ± 7.5 cm, body mass 75.0 ± 9.5 kg). Prior to participation in the experiments, all participants freely signed informed consent, which was approved by the ethics committee of the Humboldt-Universität zu Berlin (HU-KSBF-EK_2018_0005) and was in accordance with the Declaration of Helsinki. Kinematics, electromyographic activity (EMG) and the AT elongation of the right leg were measured at three speeds (transition: 2.0 m s^−1^, slow: 2.5 m s^−1^ and fast: 3.0 m s^−1^) during running on a treadmill (Daum Electronic, Ergorun Premium8, Fürth, Germany). Further, the AT force–elongation relationship of the right leg from each participant was experimentally determined with a combination of dynamometry and ultrasound imaging.

### 2.2. Measurement of kinematics and electromyographic activity

The kinematic data during barefoot running were collected using fourteen infrared cameras at 250 Hz (Vicon Motion Systems, Oxford, UK). Reflective markers (14 mm in diameter) were placed on anatomical landmarks, i.e. the tip of the second metatarsal, medial and lateral malleolus, medial and lateral epicondyles of the femur and the greater trochanter. One additional reflective foil marker (5 mm in diameter, flat surface) was placed using ultrasound guidance on the insertion of the AT at the notch of the tuber calcanei. The coordinates of the markers were filtered using a fourth-order low pass and zero-phase shift Butterworth filter with a cut-off frequency of 12 Hz. The ankle and knee joint angles were calculated in the sagittal plane using the horizontal (anteroposterior) and vertical coordinates of the reflective markers. The ankle joint angle was defined as the angle between the line crossing the tip of the second metatarsal and calcaneus marker (foot) and the line crossing the midpoints of the lateral and medial reflective markers on the malleoli and the femur condyles (shank). The knee joint angle was defined as the angle between the line crossing the lateral femur epicondyle and greater trochanter reflective marker and the shank. Both joint angles were calculated with reference to a neutral, quiet stance position. Positive angle values represent ankle plantar and knee flexed joint position. The touchdown of the foot was determined based on the heel marker's instant minimal vertical position [21]. The foot take-off was defined with regard to the first knee joint angle minimum (peak of knee extension) in the second half of the stance phase [22]*.* We calculated cadence (number of steps per second), stance and swing times, step length and duty factor (fraction of gait cycle on the ground). A wireless system (Myon m 320RX, Myon AG, Baar, Switzerland) was used to measure the EMG activity of the soleus, gastrocnemius medialis and tibialis anterior muscles at 1000 Hz. A fourth-order high-pass zero-phase shift Butterworth filter with a 50 Hz cut-off frequency, a full-wave rectification, and a low-pass zero-phase shift filter with a 20 Hz cut-off frequency were used to process the raw EMG signals. The EMG activity of soleus and gastrocnemius medialis was normalized to the highest processed EMG value acquired during a plantar flexion maximum voluntary contraction (MVC) at an ankle joint angle of 10° dorsi flexion and the tibialis anterior during a dorsiflexion MVC at an ankle joint angle of 30° plantar flexion.

### 2.3. Measurement of AT length and quantification of AT force during running

**Figure 1.**
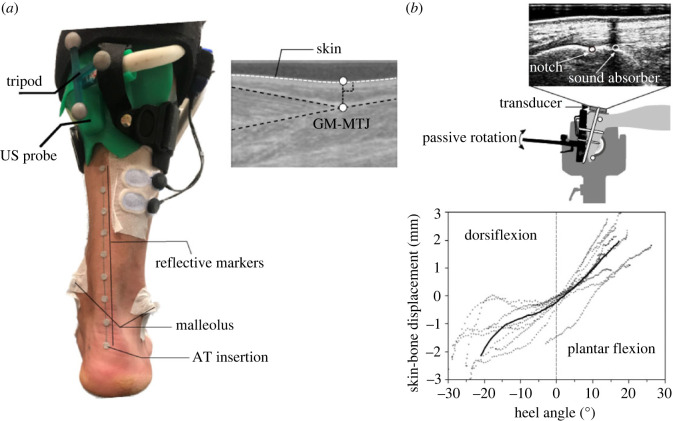
Experimental setup for the measurement of the Achilles tendon (AT) length during running. (*a*) Reflective markers were used to reconstruct the curved shape of the AT and to define the AT line of action. An ultrasound probe (US probe) was used to detect the movements of gastrocnemius medialis myotendinous junction (GM-MTJ) as the AT origin, which was then projected to the skin surface. The tripod markers were used to transfer the detected positions of the GM-MTJ to the global coordinate system. The AT lever arm was measured as the perpendicular distance from the rotation axis of the ankle joint, defined as the line connecting the malleoli, to the AT line of action. (*b*) Procedure to account for the relative movement of the calcaneus reflective marker on the skin to the bony notch at tuber calcanei. The relative displacement of the absorptive marker to the notch has been measured individually during three passive foot rotation trials from 30° plantar flexion to the individual maximum dorsiflexion using a 3 cm ultrasound transducer and computed as a function of the heel angle, which then could be applied during running. Positive heel angles represent plantar flexed and negative dorsiflexed ankle joint positions.

The methodology used for the measurement of the AT length has been reported in detail earlier [23]. Briefly, the AT curved path from the notch of the tuber calcanei (insertion of the AT) to the most distal part of the gastrocnemius medialis myotendinous junction (GM-MTJ, origin of the AT) was measured using reflective markers placed on the skin, while the GM-MTJ was registered using ultrasound (Aloka UST-5713T, Hitachi Prosound, alpha 7, Japan) at a sampling frequency of 146 Hz (figure 1*a*). The three-dimensional coordinates of the reflective markers were recorded using the motion capture system and were filtered using a fourth-order low pass and zero-phase shift Butterworth filter with a cut-off frequency of 12 Hz.

A semi-automatic tracking algorithm was implemented in a self-developed user interface (MATLAB, version 9.6, The MathWorks Inc., Natick, Massachusetts) to track the position of the GM-MTJ from the ultrasound images. The details of the developed algorithm and its validity compared with manual tracking during human walking and running (i.e. *r*^2^ = 0.97) were provided earlier [23]. The digitized coordinates of the GM-MTJ were first projected to the skin surface in the ultrasound images (figure 1*a*) and then transferred to the global, motion capture coordinate system by three transformation matrices. The first transformation matrix served to transform the coordinates of the ultrasound images to the probe coordinate system located on the protective front face of the probe and was defined by using a custom-made three-dimensional-printed calibration tool. The second transformation matrix served to transform the probe coordinates to a coordinate system defined by a triangular tripod, which was mounted on the probe. This transformation matrix was defined in a separate static calibration trial where the position of the tripod was determined relative to the defined ultrasound probe coordinate system using the motion capture system. Finally, the third transformation matrix served to transform the tripod coordinates to the global coordinate system, which was defined by capturing the coordinates of the tripod by the motion capture system during running. The curved length of the AT was determined as the sum of vectors between every two consecutive points using the three-dimensional coordinates of the markers from the calcaneus AT insertion marker to the GM-MTJ projected to the skin. In order to account for the relative movement of the calcaneus reflective marker (defining the AT insertion) to the notch of the tuber calcanei during running (i.e. skin-to-bone movement), the displacement of a sound absorptive skin marker relative to the notch was measured in a separate experiment using a 3 cm ultrasound transducer (My Lab60, Esaote, Genova, Italy, 37 Hz) (figure 1*b*).

Three trials were recorded while the foot was rotated passively (5° s^−1^) by a dynamometer (Biodex-System 3, Biodex Medical Systems Inc., USA) from 30° plantar flexion to the individual maximum dorsiflexion. The measured displacement of the absorptive skin marker relative to the notch was computed as a function of the heel angle (angle between the line crossing the calcaneus marker and the midpoint of the lateral and medial markers on the malleoli and the shank) using the motion capture system and served as a model to correct the AT length during running (figure 1*b*). Using the reconstructed curved path of the AT, the instantaneous AT lever arm was determined as the perpendicular distance from the rotation axis of the ankle joint, defined as the line connecting the lateral and medial markers of the malleoli, to the AT line of action (figure 1*a*). The line of action of the AT was defined as the midpoint of the AT, where the distance between the skin surface and the midpoint of the AT was measured using sagittal plane ultrasound scans and considered in the determination of the AT lever arm.

For establishing the individual force–elongation relationship of the AT, the ankle joint moments during five plantar flexion ramp MVCs (with a gradual increase in force exertion from rest to maximum in 5 s) from each participant were measured, with the knee extended and the ankle angle at neutral position (tibia perpendicular to the sole), using the Biodex dynamometer. Effects of gravitational forces and the misalignment of the ankle joint and dynamometer axes during the MVCs were considered using inverse dynamics [24]. The relevant kinematic data were captured by the motion capture system with nine cameras operating at 250 Hz. Further, the effect of co-activation of the antagonistic tibialis anterior muscle on the resultant joint moment during the MVCs was considered using an established EMG-based approach [25]. The AT force was calculated by dividing the ankle joint moment by the AT lever arm. A 10-cm linear ultrasound probe (My lab60, Esaote, Genova, Italy, 25 Hz) was fixed above the GM-MTJ to measure the corresponding elongation of the AT during the five MVCs. To take into consideration the effect of the inevitable joint angular rotation on the displacement of the GM-MTJ during the contractions, an additional trial with a passive rotation of the ankle joint at 5° s^−1^ was captured [24]. The resting length of the AT was defined as the length of the curved path from the marker covering the notch of the tuber calcanei to the GM-MTJ with the ankle joint set to 20° plantar flexion [26]. To achieve excellent reliability, the AT force–elongation relationship of the five ramp MVCs of each participant was averaged [27] and fitted using a quadratic function, which was then used as calibration measure to quantify the AT force during running. AT elastic strain energy was calculated as the integral of the AT force over AT elongation and the energy rate as the first time derivative of the AT energy.

### 2.4. Calculation of soleus and gastrocnemii muscles kinetics

**Figure 2.**
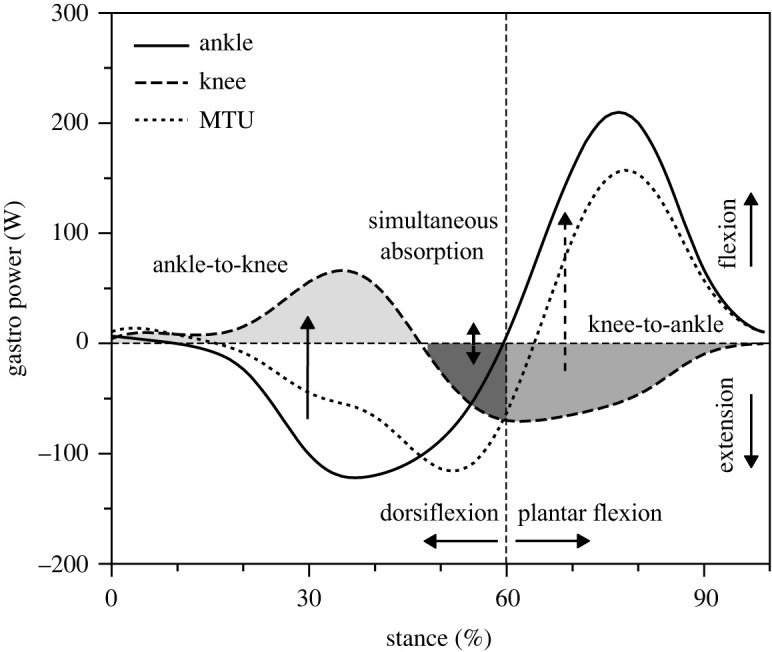
Mechanical power at the ankle and knee joints of the gastrocnemii muscles (gastro) and mechanical power of the gastrocnemii muscle–tendon unit (MTU) during the stance phase of running at 3.5 m s^−1^ running speed. The integral of the positive knee joint mechanical power of the gastrocnemii during the ankle dorsiflexion (dorsiflexion) and knee flexion (flexion) represents the ankle-to-knee joint energy transfer (ankle-to-knee). The integral of the negative knee joint mechanical power of the gastrocnemii during the ankle dorsiflexion and knee extension (extension) represents the absorbed energy at the knee joint during the simultaneous ankle/knee joint energy absorption phase (simultaneous absorption). The integral of the negative knee mechanical power of the gastrocnemii during the ankle plantar flexion (plantar flexion) and knee extension represents the knee-to-ankle joint energy transfer (knee-to-ankle).

The quantified AT force during running is generated by the three muscles of the triceps surae. The AT force contribution of each muscle was assumed to be proportional to its relative physiological cross-sectional area (PCSA) within the triceps surae, which according to Albracht *et al*. [17] is 62% for the soleus, 26% for the gastrocnemius medialis and 12% for the gastrocnemius lateralis. The moment generated by the soleus muscle at the ankle joint was calculated as the product of the soleus muscle force and the instantaneous AT lever arm measured during running. The moments at the ankle and knee joint generated by the gastrocnemius medialis and lateralis were calculated as the product of their muscle forces and their instantaneous lever arms at the ankle and knee joints. For the ankle joint, the measured AT lever arm was used. For the knee joint, the gastrocnemii lever arms as a function of the knee joint angle were extracted using the values reported by Buford *et al*. [28]. The mechanical power of the monoarticular soleus and the biarticular gastrocnemii muscles at the ankle/knee joint was calculated as the product of their ankle/knee joint moment and ankle/knee joint angular velocity. The soleus mechanical power at the ankle joint is equal to the mechanical power of the soleus muscle–tendon unit (MTU) calculated as the product of the soleus force and soleus MTU velocity [12]. The sum of the mechanical power performed at the ankle and knee joint by the two biarticular gastrocnemii equals the mechanical power performed by the gastrocnemii MTUs calculated as the product of their forces and MTU velocities [12]. The ankle/knee mechanical work performed by the soleus and the gastrocnemii muscles was then calculated as the integral of their ankle/knee joint power over time. In the analysis, the sum of the moments, power and work of the gastrocnemius medialis and lateralis was used and all muscle kinetics data will be presented as values of the gastrocnemii muscles. During the first part of the stance phase (i.e. during the simultaneous dorsi flexion and knee flexion) the mechanical power of the gastrocnemii at the knee joint was positive (figure 2), indicating an ankle-to-knee joint energy transfer. The integral of the positive knee joint mechanical power performed by the gastrocnemii quantifies the ankle-to-knee joint energy transfer. Between 45 and 60% of the stance phase, the ankle joint continued the dorsi flexion and the knee joint was extended. In this phase, the gastrocnemii absorbed energy at both joints. The integral of the negative mechanical power at the knee joint by the gastrocnemii muscles (figure 2) quantifies the absorbed energy at the knee joint during the simultaneous ankle/knee joints energy absorption phase. In the last part of the stance phase and during the simultaneous plantar flexion and knee extension, the mechanical power of the gastrocnemii at the knee joint was negative (figure 2), indicating a knee-to-ankle joint energy transfer. The integral of the negative knee mechanical power of the gastrocnemii in this phase quantifies the knee-to-ankle joint energy transfer.

### 2.5. Sensitivity analysis

Distribution coefficients proportional to the muscle relative PCSA were used for the calculation of the individual triceps surae muscle forces. However, the individual muscle force also depends on the respective force–length–velocity potential (i.e. fraction of maximum force according to the force–length and force–velocity curves) and muscle activation. Some studies that examined the soleus and gastrocnemius medialis fascicle kinetics reported force–length–velocity potentials between 0.6 and 0.8 during submaximal running [5,7,19,29]. A sensitivity analysis was performed by modifying the force–length–velocity potential of the soleus and gastrocnemius medialis muscles from 0.6 to 0.8 in 0.1 steps (i.e. nine combinations) to evaluate the effect of variable force–length–velocity potentials of the single triceps surae muscles on the main outcomes of the study (i.e. performed mechanical work of the soleus and gastrocnemii at the ankle joint and the knee-to-ankle joint energy transfer via the gastrocnemii muscles). To the authors’ knowledge, force–length–velocity potentials for the gastrocnemius lateralis muscle during running have not been reported yet, and therefore similar values as for the gastrocnemius medialis were assumed. The measured average normalized EMG activities of the soleus and gastrocnemius medialis muscles during running were used for the muscle activation. Several studies [30,31] reported similar activation patterns of gastrocnemius medialis and gastrocnemius lateralis at running speeds similar to the ones in the present study. Therefore, in the sensitivity analysis, similar activation of the two muscles was assumed. The new distribution coefficients (*k_i_*) used for the sensitivity analysis were

$$k_{i}=\frac{\left(\lambda_{i}^{l,v}\cdot \alpha_{i}\cdot r_{i}^{PCSA}\right)}{\sum_{i}^{3}\left(\lambda_{i}^{l,v}\cdot \alpha_{i}\cdot r_{i}^{PCSA}\right)},$$

with *i*: soleus, gastrocnemius medialis, gastrocnemius lateralis; $\lambda_{i}^{l,v}$: force–length–velocity potential of muscle *i*; *α_i_*: activation of muscle *i*; $r_{i}^{PCSA}$: relative PCSA of muscle *i*.

### 2.6. Statistics

In our analysis, we included nine consecutive stance phases of the right leg for each participant and averaged all investigated outcomes (i.e. temporal and spatial running parameters, kinematics, EMG, AT elongation, AT force, energy, moments, mechanical power, and work). A linear mixed model was used to test for the main effect of running speed (transition, slow, fast) on the investigated outcomes. The formula of the linear mixed model is described as follows:

$$DV\;=b_{0}+g_{\mathrm{slow}}.b_{1}+g_{\mathrm{transition}}.b_{2}+C,$$

where *DV* is the value of the dependent variable, *b*_0_ is the coefficient of the fast running speed, *b*_1_ is the coefficient of the slow running speed, *b*_1_ is the coefficient of the transition running speed, *C* is the individual intercept, *g*_slow_ is the group indicator for slow running (0 for transition running and 1 for slow running speeds) and *g*_transition_ is the group indicator for transition running (1 for transition running and 0 for slow running speeds). In the linear mixed-effects model, the participants were treated as random effects and running speed as fixed effect.

A pairwise Tukey test was performed as a *post hoc* analysis in the case of a significant main effect of speed, and Benjamini–Hochberg corrected *p*-values are reported. The statistical analyses were conducted using R v4.0.1 (R Foundation for Statistical Computing, Vienna, Austria), where the ‘nlme’ package was used for the linear mixed model and the ‘emmeans’ package for *post hoc* testing. A one-way ANOVA for repeated measures was performed with speed as within-subject factors to compare the outcomes between the soleus and the gastrocnemii muscles. The significance level was set to *α* = 0.05, and all values are reported as mean ± standard errors.

## 3. Results

**Figure 3.**
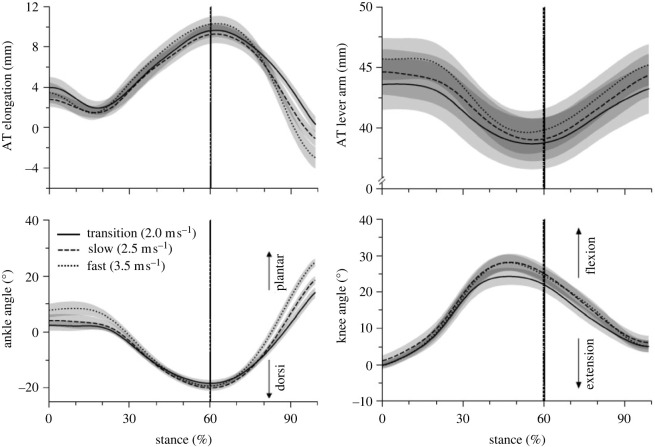
Achilles tendon (AT) elongation, AT lever arm, ankle and knee joint angles during the stance phase of running at transition (2.0 m s^−1^), slow (2.5 m s^−1^) and fast (3.5 m s^−1^) running speed. The vertical lines separate the dorsiflexion and plantar flexion of the ankle joint. The curves and shaded areas represent mean ± standard errors.

**Figure 4.**
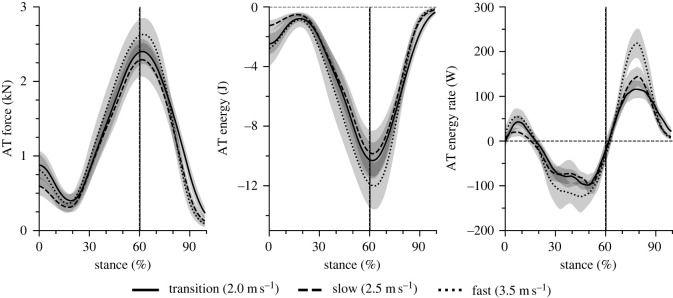
Achilles tendon (AT) force, AT energy and rate of AT energy during the stance phase of running at transition (2.0 m s^−1^), slow (2.5 m s^−1^) and fast (3.5 m s^−1^) running speed. The vertical lines separate the dorsiflexion and plantar flexion of the ankle joint. The curves and shaded areas represent mean ± standard errors.

**Figure 5.**
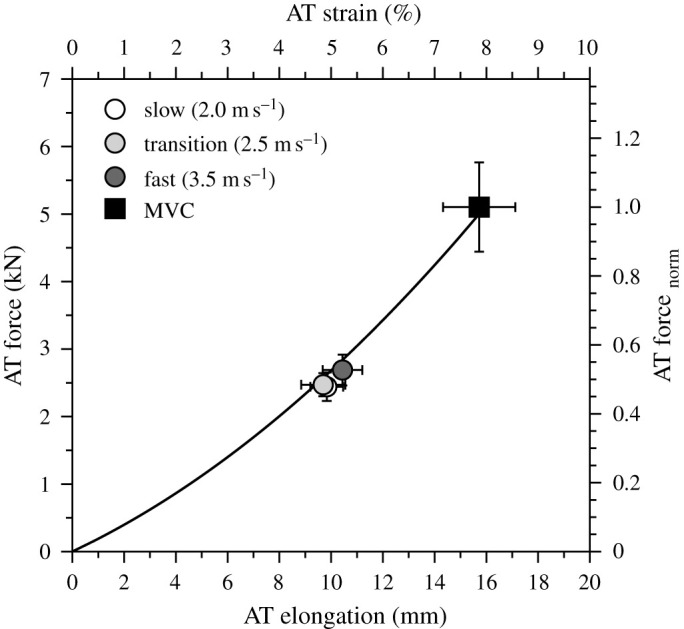
Force elongation relationship of the Achilles tendon (AT) during maximum voluntary plantar flexion contractions (MVC) and the operating maximum AT force and elongation values during the stance phase of running at transition (2.0 m s^−1^), slow (2.5 m s^−1^) and fast (3.5 m s^−1^) running speed. The top horizontal axis shows the AT strain and the right vertical axis shows the AT tendon force normalized to maximum AT force during the MVC. All values are presented as mean ± standard error.

**Figure 6.**
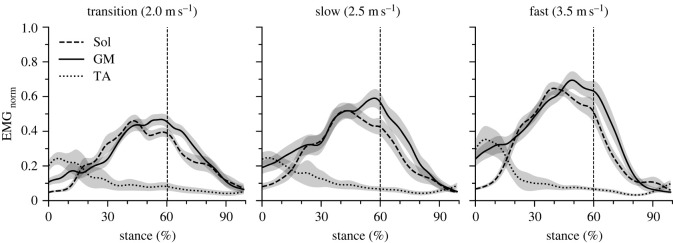
Electromyographic activity of the soleus (Sol), gastrocnemius medialis (GM) and tibialis anterior (TA) muscles normalized to maximum voluntary isometric contractions (EMG_norm_) during the stance phase of running at transition (2.0 m s^−1^), slow (2.5 m s^−1^) and fast (3.5 m s^−1^) running speed. The vertical lines separate the dorsiflexion and plantar flexion of the ankle joint. The curves and shaded areas represent mean ± standard errors.

Cadence and step length increased significantly as a function of running speed (table 1; *p* < 0.001). Stance time and duty factor were reduced significantly (*p* < 0.001); however, the swing time did not change (*p* = 0.122) with increasing running speed (table 1). The maximum force applied to the AT during the isometric ramp MVC was 5103 ± 627 N and the maximum AT elongation 15.7 ± 1.4 mm. Figure 3 shows the AT elongation, AT lever arm, ankle and knee joint angles and figure 4 the AT force, AT energy and rate of the AT energy during the stance phase of running. Maximum AT elongation (*p* = 0.567) and AT force (*p* = 0.335) as well as AT energy recoil (*p* = 0.101) did not show a significant speed effect, though the rate of AT energy recoil increased with speed (table 2). The maximum AT force at the three investigated running speeds was on average approximately 50% of the achieved maximum AT force during the isometric MVC (figure 5). Running speed influenced the maximum EMG activity of the soleus (*p* < 0.001) and gastrocnemius medialis (*p* < 0.001) muscles (figure 6). Both muscles increased their EMG activity with increasing running speed (table 2). The maximum EMG activity of the tibialis anterior did not show a significant speed effect (*p* = 0.394; table 2).

**Table 1.** Spatio-temporal parameters in the investigating running speeds. All values are presented as mean ± standard error. *Statistically significant effect of speed (*p* < 0.05). Each row sharing the same letter does not differ significantly (*p* > 0.05, *post hoc* analysis).

running speed	transition (2.0 m s^−1^)	slow (2.5 m s^−1^)	fast (3.5 m s^−1^)
cadence (steps s^−1^) *	2.61 ± 0.04 **a**	2.71 ± 0.05 **b**	2.92 ± 0.05 **c**
step length (m) *	0.76 ± 0.01 **a**	0.92 ± 0.02 **b**	1.20 ± 0.02 **c**
stance time (ms) *	348 ± 10 **a**	325 ± 13 **b**	283 ± 6 **c**
swing time (ms)	418 ± 8	416 ± 10	403 ± 9
duty factor *	0.45 ± 0.01 **a**	0.44 ± 0.01 **a**	0.41 ± 0.01 **b**

**Table 2.** Maximum Achilles tendon (AT) elongation and AT force as well as AT energy recoil, rate of the AT energy recoil and normalized maximum electromyographic activity of the soleus (Sol), gastrocnemius medialis (GM) and tibialis anterior (TA) muscles for the investigated running speeds. All values are presented as mean ± standard error. *Statistically significant effect of speed (*p* < 0.05). Each row sharing the same letter does not differ significantly (*p* > 0.05, *post hoc* analysis).

running speed	transition (2.0 m s^−1^)	slow (2.5 m s^−1^)	fast (3.5 m s^−1^)
AT elongation_max_ (mm)	9.8 ± 0.6	9.7 ± 0.8	10.4 ± 0.8
AT force_max_ (N)	2471 ± 171	2441 ± 210	2690 ± 228
AT energy (J)	10.4 ± 1.2	10.5 ± 1.4	12.3 ± 1.6
AT energy rate (W) *	147.2 ± 17.2 **a**	181.8 ± 20.5 **b**	269.7 ± 28.9 **c**
Sol *	0.56 ± 0.03 **a**	0.66 ± 0.04 **b**	0.77 ± 0.04 **c**
GM *	0.52 ± 0.04 **a**	0.75 ± 0.04 **b**	0.80 ± 0.05 **b**
TA	0.34 ± 0.07	0.38 ± 0.06	0.45 ± 0.08

**Figure 7.**
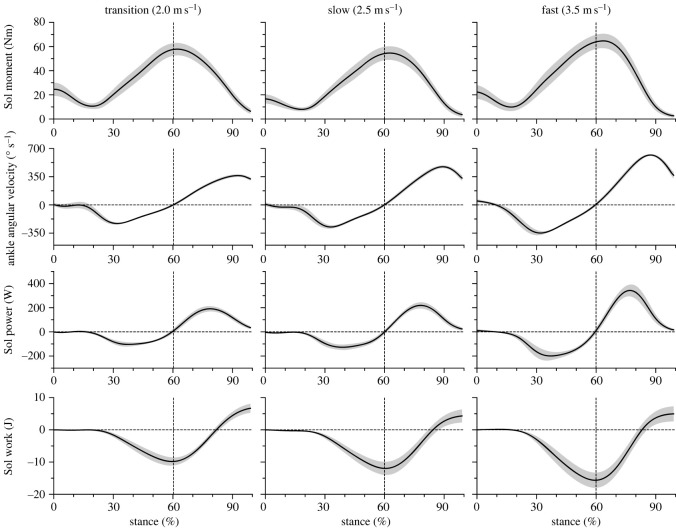
Moment, angular velocity, mechanical power and work at the ankle joint of the soleus muscle (Sol) during the stance phase of running at transition (2.0 m s^−1^), slow (2.5 m s^−1^) and fast (3.5 m s^−1^) running speed. Negative values in the mechanical power indicate energy absorption during dorsiflexion and positive values energy production during plantar flexion at the ankle joint. The vertical lines separate the dorsiflexion and plantar flexion of the ankle joint. The curves and shaded areas represent mean ± standard errors.

The maximum moment generated by the monoarticular soleus muscle at the ankle joint was not affected by running speed (*p* = 0.406; figure 7, table 3). The minimum and maximum mechanical power of the soleus at the ankle joint demonstrated a significant speed effect (*p* < 0.001) with a continuous increase from transition to fast running speed (figure 7, table 3). Speed significantly influenced the negative (*p* = 0.030) and positive (*p* = 0.033) mechanical work of the soleus muscle at the ankle joint (figure 7). *Post hoc* comparisons showed that the negative and positive work of the soleus were greater in fast compared to the transition and slow speed (table 3). The net mechanical work of the soleus at the ankle joint was invariant over running speeds (table 3).

**Table 3.** Maximum moment, minimum/maximum mechanical power, negative/positive mechanical work and net work of the soleus (Sol) muscle at the ankle joint. All values are presented as mean ± standard error. *Statistically significant effect of speed (*p* < 0.05). Each row sharing the same letter does not differ significantly (*p* > 0.05, *post hoc* analysis).

running speed	transition (2.0 m s^−1^)	slow (2.5 m s^−1^)	fast (3.5 m s^−1^)
Sol ankle moment_max_ (Nm)	59.3 ± 5.0	58.6 ± 5.3	66.0 ± 6.1
Sol ankle power_min_ (W) *	−120.6 ± 15.4 **a**	−159.7 ± 24.1 **b**	−251.3 ± 38.9 **c**
Sol ankle power_max_ (W) *	205.2 ± 22.2 **a**	236.6 ± 21.3 **b**	380.7 ± 44.9 **c**
Sol negative ankle work (J) *	−10.2 ± 1.3 **a**	−12.3 ± 2.0 **a**	−16.0 ± 2.4 **b**
Sol positive ankle work (J) *	16.9 ± 2.2 **a**	16.6 ± 2.0 **a**	21.0 ± 3.3 **b**
Sol net ankle work (J)	6.7 ± 1.4	4.3 ± 2.0	4.7 ± 2.3

**Figure 8.**
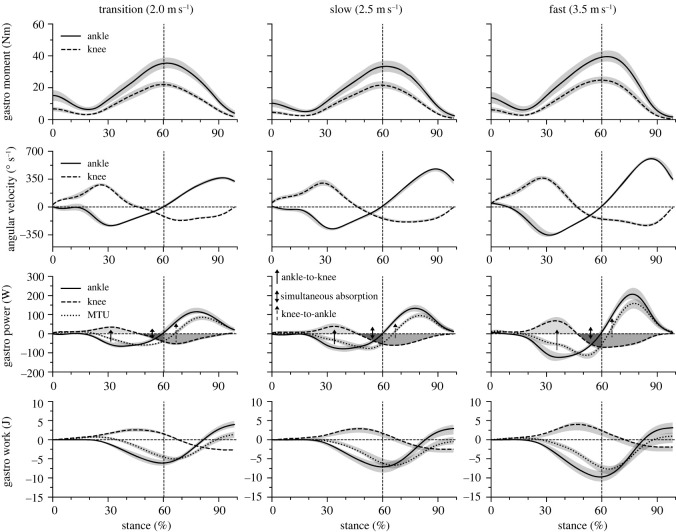
Moment, angular velocity, mechanical power and work at the ankle and knee joints of the gastrocnemii muscles (gastro) and mechanical power of the gastrocnemii muscle–tendon unit (MTU) during the stance phase of running at transition (2.0 m s^−1^), slow (2.5 m s^−1^) and fast (3.5 m s^−1^) running speed. Negative values in the mechanical power indicate energy absorption at the ankle joint during dorsiflexion, energy absorption at the knee joint during knee extension and energy absorption of the muscle–tendon unit during lengthening. Positive power values indicate energy production during ankle plantar flexion, knee flexion and MTU shortening. The shaded areas and arrows (third row) indicate the transferred energy from ankle to knee joint during the first part of the stance phase (ankle-to-knee), the absorbed energy at the knee joint during the simultaneous ankle/knee joints energy absorption phase (simultaneous absorption) and the transferred energy from knee to ankle joint during the push-off phase of running (knee-to-ankle). The vertical lines separate the dorsiflexion and plantar flexion of the ankle joint. The curves and shaded areas represent mean ± standard errors.

**Figure 9.**
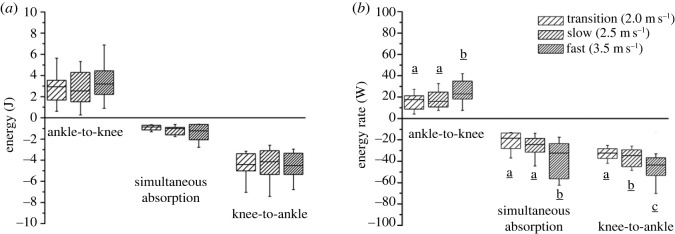
(*a*) Transferred energy from ankle to knee joint during the first part of the stance phase (ankle-to-knee), absorbed energy at the knee joint during the simultaneous ankle/knee joints energy absorption phase (simultaneous absorption) and transferred energy from knee to ankle joint during the push-off phase of running (knee-to-ankle). (*b*) Rate of the ankle-to-knee energy transfer, rate of the absorbed energy during the simultaneous ankle/knee joint energy absorption phase and rate of knee-to-ankle energy transfer during the stance phase of running. Each boxplot sharing the same letter among the three phases does not differ significantly (*p* > 0.05, *post hoc* analysis).

The maximum moments of the biarticular gastrocnemii at the ankle and knee joint did not show a significant speed effect (*p* = 0.407 and *p* = 0.192) (figure 8, table 4). The mechanical power of the gastrocnemii muscles at the ankle and knee joints (figure 8) demonstrated three characteristic phases during the stance phase of running, where the biarticular mechanisms of the gastrocnemii can influence the mechanical power and work at the ankle joint, separate from their own musculotendinous power and work production. In the first part of the stance phase (approx. 45% of stance), the mechanical power of the gastrocnemii at the ankle and knee joint had opposite signs. In this phase, energy was transferred from the ankle to the knee joint. Between 45 and 60% of the stance phase, the gastrocnemii simultaneously absorbed energy at both joints, thus distributing their mechanical power and work between the two joints. In the last part of the stance phase (greater than 60%), the mechanical power of the gastrocnemii at the ankle and knee joint had opposite signs again. In this phase, energy was transferred from the knee to the ankle joint. The ankle-to-knee joint energy transfer (*p* = 0.300), the energy absorbed at the knee joint during the simultaneous ankle/knee joint energy absorption phase (*p* = 0.235) and the knee-to-ankle joint energy transfer (*p* = 0.495) did not show any significant speed effect (figure 9). The energy rate in the three characteristic phases, where the biarticular mechanisms were involved, was significantly affected by speed (*p* < 0.05). The rate of the knee-to-ankle joint energy transfer continuously increased with increasing running speed (*p* < 0.05), whereas in the two other phases the energy rates were significantly (*p* < 0.05) higher in the fast compared to the transition and slow speeds (figure 9).

**Table 4.** Maximum moment and minimum/maximum mechanical power of the gastrocnemii muscles (gastro) at the ankle/knee joint and the gastrocnemii muscle–tendon unit (MTU). All values are presented as mean ± standard error. *Statistically significant effect of speed (*p* < 0.05). Each row sharing the same letter does not differ significantly (*p* > 0.05, *post hoc* analysis).

running speed	transition (2.0 m s^−1^)	slow (2.5 m s^−1^)	fast (3.5 m s^−1^)
gastro ankle moment_max_ (Nm)	36.3 ± 3.1	35.9 ± 3.2	40.4 ± 3.7
gastro knee moment_max_ (Nm)	22.7 ± 1.8	22.9 ± 2.2	25.5 ± 2.4
gastro ankle power_min_ (W) *	−73.8 ± 9.4 **a**	−97.8 ± 14.7 **b**	−154.1 ± 23.8 **c**
gastro ankle power_max_ (W) *	125.8 ± 13.6 **a**	145.0 ± 13.1 **b**	233.3 ± 27.5 **c**
gastro knee power_min_ (W) *	−61.2 ± 6.6 **a**	−68.7 ± 6.9 **a**	−85.2 ± 12.0 **b**
gastro knee power_max_ (W) *	37.2 ± 6.6 **a**	46.8 ± 9.2 **a**	79.1 ± 22.2 **b**
gastro MTU power_min_ (W) *	−77.1 ± 6.5 **a**	−92.7 ± 11.5 **b**	−134.5 ± 13.3 **c**
gastro MTU power_max_ (W) *	95.3 ± 12.0 **a**	105.3 ± 10.5 **b**	176.6 ± 23.5 **c**

Speed significantly affected the minimum and maximum mechanical power of the gastrocnemii muscles at the ankle (*p* < 0.001) and knee joint (*p* = 0.020; table 4). At the ankle joint, the mechanical power increased as a function of speed, while at the knee joint the values were significantly greater in the fast compared to transition and slow speeds (table 4). The minimum and maximum mechanical power of the gastrocnemii MTU also increased with speed (*p* < 0.001; table 4). Speed influenced the negative (*p* = 0.029) and positive (*p* = 0.030) mechanical work at the ankle joint by the gastrocnemii muscles (figure 8, table 5). *Post hoc* comparisons showed higher values in the negative (*p* = 0.028) and positive (*p* = 0.030) mechanical work in the fast compared to the transition and slow speeds. At the knee joint, both the negative and positive mechanical work did not show significant differences with increasing speed (*p* = 0.357 and *p* = 0.300; table 5). A speed effect on the negative and positive mechanical work by the gastrocnemii MTU was found (*p* = 0.023) with higher values in the fast compared to the transition and slow speeds (table 5). The net mechanical work by the biarticular gastrocnemii at the ankle and knee joint and the network of the gastrocnemii MTU were not significantly influenced by speed (table 5). The ankle joint maximum moment, maximum mechanical power and network of the soleus were significantly (*p* < 0.001) higher compared to the gastrocnemii muscles (tables 3, 4 and 5).

**Table 5.** Negative, positive and net work of the gastrocnemii muscles (gastro) at the ankle/knee joint and the gastrocnemii muscle–tendon unit (MTU). All values are presented as mean ± standard error. *Statistically significant effect of speed (*p* < 0.05). Each row sharing the same letter does not differ significantly (*p* > 0.05, *post hoc* analysis).

running speed	transition (2.0 m s^−1^)	slow (2.5 m s^−1^)	fast (3.5 m s^−1^)
gastro negative ankle work (J) *	−6.3 ± 0.8 **a**	−7.5 ± 1.2 **a**	−9.8 ± 1.4 **b**
gastro positive ankle work (J) *	10.4 ± 1.3 **a**	10.2 ± 1.3 **a**	12.9 ± 1.9 **b**
gastro net ankle work (J)	4.1 ± 0.9	2.6 ± 1.2	3.0 ± 1.4
gastro negative knee work (J)	−5.5 ± 0.5	−5.6 ± 0.6	−6.2 ± 0.8
gastro positive knee work (J)	2.8 ± 0.5	2.9 ± 0.6	4.1 ± 1.1
gastro net knee work (J)	−2.7 ± 0.2	−2.7 ± 0.5	−2.1 ± 0.6
gastro negative MTU work (J) *	−5.3 ± 0.5 **a**	−6.6 ± 1.0 **a**	−8.0 ± 0.9 **b**
gastro positive MTU work (J) *	6.7 ± 1.0 **a**	6.4 ± 0.9 **a**	8.8 ± 1.6 **b**
gastro net MTU work (J)	1.4 ± 0.8	−0.1 ± 0.9	0.8 ± 1.3

Table 6 shows the results of the sensitivity analysis considering the positive ankle mechanical work of the soleus and gastrocnemii muscles as well as the knee-to-ankle joint energy transfer during the push-off phase of running. Represented are the combinations of the force–length–velocity potential of the soleus and gastrocnemii muscles with the largest and lowest positive ankle mechanical work from the soleus. Different force potentials affect the absolute values in all three parameters and influence the relative contribution of the triceps surae muscles to the ankle's mechanical work. Nevertheless, in all combinations the contribution of the soleus was higher than the gastrocnemii. Combinations with a lower force–length–velocity potential of the gastrocnemii compared to the soleus decreased the contribution of the knee-to-ankle joint energy transfer to the whole ankle mechanical work, yet it remained higher than 14% in all combinations. On the other hand, higher values in the force–length–velocity potential of the gastrocnemii compared to the soleus increased the contribution of the knee-to-ankle joint energy transfer up to 20%.

**Table 6.** Range of the positive ankle joint work from the soleus (sol) and the gastrocnemii (gastro) muscles as well as the knee-to-ankle joint energy transfer resulting from the sensitivity analysis. Lower and upper limits represent the combinations with the minimum and maximum values of the soleus positive mechanical work at the ankle joint. The values in parentheses show the percentage contribution of the knee-to-ankle joint energy transfer to the whole ankle joint work. All values are presented as mean ± standard error.

	sol positive ankle work (J)	gastro positive ankle work (J)	knee-to-ankle joint energy (J)
transition (2.0 m s^−1^)			
lower limit	14.7 ± 2.3	12.6 ± 0.7	−5.2 ± 0.51 (19%)
upper limit	18.3 ± 2.6	9.0 ± 0.5	−3.8 ± 0.39 (14%)
slow (2.5 m s^−1^)			
lower limit	14.3 ± 1.7	12.5 ± 1.6	−5.3 ± 0.7 (20%)
upper limit	18.0 ± 2.1	8.8 ± 1.2	−4.0 ± 0.5 (15%)
fast (3.5 m s^−1^)			
lower limit	17.7 ± 3.3	16.2 ± 2.2	−6.5 ± 0.6 (19%)
upper limit	22.3 ± 4.0	11.6 ± 1.5	−4.8 ± 0.4 (14%)

## 4. Discussion

The current study investigated the contribution of the monoarticular soleus and the biarticular gastrocnemii muscles to the mechanical power and work performed at the ankle joint during running. Although the contribution of the soleus muscle was greater, biarticular mechanisms of the gastrocnemii accounted for a relevant part of the mechanical power and work performed at the ankle joint. A knee-to-ankle joint energy transfer via the gastrocnemii muscles contributed to the increase of power and work production at the ankle joint during the push-off phase. Furthermore, the rate of the knee-to-ankle joint energy transfer increased with running speed, indicating a speed-related enhancement of the biarticularity effects on the power production at the ankle joint. The energy transferred from the knee to the ankle joint did not differ significantly between the three investigated speeds and was on average 41% of the total musculotendinous work of the gastrocnemii muscles at the ankle joint and accordingly 16% of the ankle joint work performed by both the soleus and gastrocnemii muscles, which shows the relevant contribution of biarticular mechanisms to the required ankle joint mechanical power and work during running. The applied sensitivity analysis demonstrated that these findings generally hold true with respect to variations of the force–length–velocity potentials of the individual triceps surae muscles, thus with force sharing among the three synergistic muscles.

With an increase of running speed, there was a decrease in stance time and duty factor and an increase in step length, cadence and in the EMG activity of the soleus and gastrocnemius medialis muscles. The maximum AT forces in the three running speeds were on average between 2.471 and 2.690 kN, corresponding to approximately 50% of the maximum AT force (5.103 kN) generated during the isometric MVC. Recent studies investigating the operating length and velocity of the soleus and gastrocnemius medialis fascicles reported a force–length–velocity potential between 0.60 and 0.80 of the maximum muscle force during the stance phase at similar running speeds [5,7,19,29]. The measured maximum normalized EMG activities of the soleus and gastrocnemius medialis muscles were on average approximately 70%. Adopting a Hill-type muscle model—i.e. assuming that muscle force is equal to the product of the muscle force–length–velocity potential, activation and maximum isometric muscle force—AT forces between 2.143 and 2.857 kN were predicted, which are very close to the quantified AT forces.

The voluminous soleus muscle performed more mechanical power and work at the ankle joint compared to the gastrocnemii muscles. Also, the net mechanical work of the soleus muscle at the ankle joint was greater than the net work of the gastrocnemii. The increased power at the ankle joint by the monoarticular soleus during the push-off phase with increasing speed can be affected by a modulation of the rate of elastic energy recoil of the soleus tendinous structures and the mechanical power of the soleus contractile element. The increased rate of AT energy recoil with increasing running speed is evidence for the contribution of soleus elastic structures to the increased mechanical power at the ankle joint during the push-off phase. However, the positive net ankle work of the soleus muscle during running can only be the result of the soleus contractile element, as the elastic element cannot produce positive net work. The gastrocnemii muscles on the other hand can generate moments, power and work around the two joints that are spanned. Therefore, biarticular mechanisms can influence, separately from their own musculotendinous power/work output, the performed power and work at the ankle joint. The relative contribution of the biarticular mechanisms to the net ankle mechanical work of the gastrocnemii muscles was on average 76%, indicating a higher involvement of the biarticularity compared to their contractile elements. Similarly, the maximum mechanical power of the gastrocnemii muscles at the ankle joint was on average 34% greater than the maximum mechanical power of the gastrocnemii MTU due to the knee-to-ankle joint energy transfer. These findings show that the energy and power output of the biarticular gastrocnemii at the two spanned joints is modulated towards an increased output at the ankle joint during running.

In the first part of the stance phase and during the simultaneous dorsiflexion and knee flexion, energy was transferred from the ankle to the knee joint via the gastrocnemii muscles. In the second part of the stance and during the simultaneous plantar flexion and knee extension, energy was transferred from the knee to the ankle joint. The knee-to-ankle joint energy transfer significantly enhanced the mechanical power at the ankle joint during the push-off phase of running. This phenomenon is well known, particularly during explosive leg extensions as for example maximum vertical jumping and accelerated sprint running, where high power at the ankle joint during the last part of the push-off phase is required [9,13,14]. The elegance of the knee-to-ankle joint energy transfer mechanism lies within the principle that mechanical work production of the large monoarticular vasti muscles can be effectively transferred via the biarticular gastrocnemii to the ankle joint, where it would be disadvantageous to have large muscles due to the moment of inertia [8,10]. Although contractile energy transfer from the monoarticular vasti to the ankle joint may enhance performance in explosive leg extension tasks [10,13,14] it is metabolically costly. Due to their greater muscle volume compared to the triceps surae [15,16], the vasti can produce more muscular power and work. However, due to their longer fibres, a unit of force generation is metabolically more expensive compared to the shorter triceps surae muscles [18,19].

The amount of energy transferred from the knee to the ankle joint was on average 16% of the performed work from both soleus and gastrocnemii muscles at the ankle joint. The sensitivity analysis with a wide range of possible operating lengths and velocities of the soleus and gastrocnemii muscles resulted in a contribution of the knee-to-ankle joint energy transfer between 14 and 20%. Furthermore, the rate of the knee-to-ankle joint energy transfer increased with running speed. Therefore, one may suggest that the knee-to-ankle joint energy transfer during submaximal running with the participation of the vasti muscles increases the energetic cost of a task where the metabolic cost is more important than maximum performance. However, during the stance phase of submaximal running, the fascicles of the vastus lateralis contract nearly isometrically and very close to the optimal fascicle length for force generation, despite the lengthening–shortening behaviour of the vastus lateralis MTU [19,32]. The almost isometric contraction of the vastus lateralis indicates negligible contractile work production from the monoarticular vasti during the knee extension and an almost elastic energy recoil from the patellar-quadriceps tendon during the knee-to-ankle joint energy transfer phase. Furthermore, it indicates that the main part of the energy transferred from ankle to knee joint in the beginning of the stance phase has been stored as strain energy in the patellar-quadriceps tendon. In the ankle-to-knee joint energy transfer phase, the knee flexion moments of the gastrocnemii muscles must be counteracted by the quadriceps muscles, mainly by the monoarticular vasti due to their higher muscle volume (approx. 5.7 times) compared to rectus femoris [33]. In this phase, the knee joint undergoes a flexion (i.e. lengthening of the vasti MTU), thus the energy transferred from the ankle to the knee joint will not be absorbed by the contractile elements of the vasti and the main part will be stored as elastic energy in its tendinous tissues. The ankle-to-knee joint energy transfer was on average 30% of the AT energy recoil, thus an important energy source in running.

The findings discussed above demonstrate that the energy transfer from the ankle to the knee joint and vice versa by the biarticular gastrocnemii muscles during the stance phase of submaximal running reflects an energy exchange between elastic tissues, with negligible contractile energy absorption/production from the large proximal vasti. Considering that the vastus lateralis is operating close to the optimal length during the stance phase of running—thus with a high force–length–velocity potential, which is beneficial for economical force generation [19,32]—it can be suggested that the energy transfer between the large monoarticular vasti and the less voluminous triceps surae muscles via the biarticular gastrocnemii actually promotes running economy. A third relevant phase of stance (45 to 60% of stance) has been identified, where the ankle joint continued to dorsiflex and the knee joint extended. During this phase, the gastrocnemii absorbed power and energy in both the ankle and knee joint. Nevertheless, the AT elongated further and the AT elastic strain energy continued to increase. This type of behaviour indicates that some returned energy from the patellar-quadriceps tendon due to the shortening of the vasti MTU in this phase is transferred via the gastrocnemii to the AT and recoiled in the push-off phase. This interpretation is consistent with earlier suggestions that some work production by the quadriceps muscles might be absorbed by the gastrocnemii series elastic elements and stored as elastic energy [31].

The quantification of AT force *in vivo* is challenging and there are some limitations associated with the approach used in the present study that need to be considered when interpreting the results. The AT force–elongation relationship, determined during isometric MVCs, was used as a calibration measure to quantify the AT force during running. Although the triceps suae muscles generate the main part of the ankle joint moment during a plantar flexion MVC, also other plantar flexors contribute to the measured ankle joint moment. This means that the forces calculated for determining the AT force–elongation relationship are slightly overestimated, which also affects the AT force values determined for running. The PCSA of the triceps surae accounts for approximately 80% of the overall plantar flexor muscle PCSA [34] and the AT moment arm is on average approximately 3.7-fold larger compared to moment arm of the other plantar flexors [35,36], which indicates a negligible effect for the main messages concerning the contribution of the monoarticular soleus and the biarticular gastrocnemii to the mechanical power and work at the ankle joint during running. The rate of the AT force development during the MVCs (1.08 ± 0.46 kN s^−1^) was considerably lower than the rate of AT force development during running (17 to 20 kN s^−1^). Differences in the loading rate between the isometric MVCs and running conditions may influence the accuracy of the AT force quantification because tendons, as biomaterials, show viscoelastic properties. However, Ker [37] reported that a variation of loading rates from 0.6 to 31.2 kN s^−1^ does not affect the tendon's Young’s modulus and, therefore, we can assume a negligible effect of loading rate on tendon dynamics during running. The anatomical structure of the AT comprises bundles of fascicles originating from the three single triceps surae muscles [38]. This structure allows a certain degree of inter-fascicle sliding, which may result in different regional displacement gradients within the AT during loading [39]. Triceps surae muscle dynamics may affect the inter-fascicle sliding within the AT during running, which would affect the accuracy of our AT force quantification. On the other hand, there is also evidence that the myotendinous structures of the triceps surae MTUs do not work independently but are mechanically connected [40,41]. Myotendinous force transmission through contiguous extramuscular connective tissue structures influences the mechanical linkage of adjacent MTUs during muscle contraction [42,43] and promotes a synchronous movement of connective tissues between the triceps surae muscles [40,41]. The interfascicular matrix in tendons is quite elastic and has a considerable stiffness (approx. 50% of the tendon fascicles [44]), thus inter-fascicle sliding could strain the interfascicular matrix and facilitate force transmission within the tendon [45]. Force transmission mechanisms through the connective tissue network of the MTUs may redistribute the forces within the AT in order to minimize peak stresses [45] and thus heterogeneous regional displacement gradients. Furthermore, the twisted structure of tendons, such as the AT, functionally reduces heterogeneous strain patterns across the tendon [46]. The whole curved path of the AT from the notch of the tuber calcanei to the most distal part of the GM-MTJ was measured to quantify AT force during running. The same length was also used to measure the AT elongation during the calibration MVC trials in order to establish the individual AT force–elongation relationship. From a theoretical point of view, measurements of the whole tendon length increase the robustness of the AT force quantification. Model predictions indicate that the mean stress of the whole AT is less sensitive to morphological variations like shape or twist compared to local peak stress values [47]. Consequently, although a certain degree of heterogeneous displacement within the AT length is possible during running, which may to some extent influence the accuracy of the AT force quantification, it is unlikely to change the main findings and conclusions.

In summary, at submaximal 2.0 to 3.5 m s^−1^ running speeds, the power/work performed by the triceps surae muscles at the ankle joint increased with increasing speed. The performed power and work of the monoarticular soleus were higher compared to the gastrocnemii, yet biarticular mechanisms of the gastrocnemii muscles significantly enhanced the mechanical power and work at the ankle joint. In the current study, an ankle-to-knee joint energy transfer in the first part and a knee-to-ankle joint energy transfer in the last part of the stance phase was found via the biarticular gastrocnemii. This energy transfer between the two joints probably occurs with negligible energy absorption/production of the vasti contractile elements, thus rather through the involvement of their elastic elements. The current study improves the current understanding of how mono- and biarticular mechanisms facilitate the increase of mechanical power and work at the ankle joint during submaximal running speeds. This knowledge may be useful for designing exercise interventions and bioinspired exoskeletons for customized assistance in healthy and pathological conditions.

## Data Availability

The original data of the study can be accessed at https://doi.org/10.17605/OSF.IO/WDHR3 [48].
